# New insights into the pathogenesis of necrotizing enterocolitis and the dawn of potential therapeutics

**DOI:** 10.1016/j.sempedsurg.2023.151309

**Published:** 2023-06-01

**Authors:** Daniel J. Scheese, Chhinder P. Sodhi, David J. Hackam

**Affiliations:** 1 Division of Pediatric Surgery, Johns Hopkins University School of Medicine, Baltimore, MD, USA

## Abstract

Necrotizing enterocolitis (NEC) is a devastating gastrointestinal disorder in premature infants that causes significant morbidity and mortality. Research efforts into the pathogenesis of NEC have discovered a pivotal role for the gram-negative bacterial receptor, Toll-like receptor 4 (TLR4), in its development. TLR4 is activated by dysbiotic microbes within the intestinal lumen, which leads to an exaggerated inflammatory response within the developing intestine, resulting in mucosal injury. More recently, studies have identified that the impaired intestinal motility that occurs early in NEC has a causative role in disease development, as strategies to enhance intestinal motility can reverse NEC in preclinical models. There has also been broad appreciation that NEC also contributes to significant neuroinflammation, which we have linked to the effects of gut-derived pro-inflammatory molecules and immune cells which activate microglia in the developing brain, resulting in white matter injury. These findings suggest that the management of the intestinal inflammation may secondarily be neuroprotective. Importantly, despite the significant burden of NEC on premature infants, these and other studies have provided a strong rationale for the development of small molecules with the capability of reducing NEC severity in pre-clinical models, thus guiding the development of specific anti-NEC therapies. This review summarizes the roles of TLR4 signaling in the premature gut in the pathogenesis of NEC, and provides insights into optimal clinical management strategies based upon findings from laboratory studies.

## Graphical Abstract

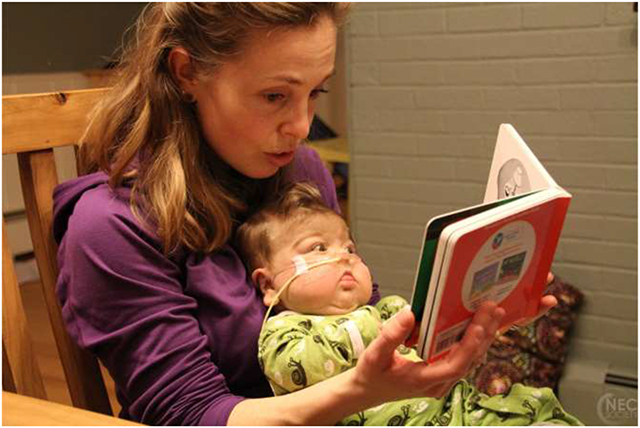

Micah and his twin were born at 27-weeks gestation. When they were six weeks old, the twins weighed five pounds, were beginning to breastfeed, and it looked like they would be discharged soon. But Micah developed necrotizing enterocolitis, which required multiple bowel resections, and he went into renal failure. Micah made it home, and his family believed their son had made it, even though he would need a kidney transplant. Tragically, Micah was readmitted to the PICU and died in his parents’ arms, from complications of NEC, just before the twins’ first birthday.

## Introduction

Despite decades of progress in the care of neonates, the development of necrotizing enterocolitis (NEC) persists as a major threat to the lives of premature infants. Along with the devastating clinical manifestations of NEC, the impact of this disease extends far beyond the individual child and is worsened by the reality that most families affected by NEC are generally unaware of its very existence until the moment of diagnosis. Accordingly, NEC often invites feelings of well-placed fear and uncertainty on the part of the parents, and misplaced guilt on the part of the medical team. From the perspective of the families, their immediate questions are “what caused NEC in the first place”, “is my baby suffering from just a bad infection”, and “did I do anything wrong”. In a strange sense of medical irony, the medical team often has the very same questions themselves, although phrased with an added layer of clinical sophistication, such as, “what is the pathogenesis of NEC”, “what is the role of the microbiome in its development”, and “were there preventable risk factors that lead to the disease”. Rarely are caregivers and patients so unexpectedly and unwillingly aligned in their appreciation of what still remains to be learned about a given disease. This review will seek to answer these questions and in doing so we will provide insights into the pathogenesis of NEC, while also discussing potential novel therapies.

### Clinical presentation of NEC

The typical presentation of a baby with NEC is that of a premature infant who is otherwise stable in the neonatal intensive care unit (NICU), and who – seemingly without warning – develops the combination of abdominal distention, emesis, and bloody stools (1). More likely than not, infants will have been fed a diet containing infant formula, which has been demonstrated to cause higher rates of NEC than breast milk feeds in various studies (2, 3). The initial approach to management of patients with NEC consists of the immediate cessation of oral feeds, intravenous fluid resuscitation, and administration of broad-spectrum antibiotics (4), which will result in the resolution of symptoms in approximately 50% of cases (5-7). The remainder of patients will experience clinical deterioration, with progression towards the development of systemic sepsis and cardiovascular collapse, reflecting worsening intra-abdominal inflammation and the development of necrosis of the small or large intestine (8). Without urgent surgery to remove the necrotic intestine, many patients who do not respond to initial medical measures will die (5). The clinical presentation of NEC has been classified according to the Bells staging system, which was first described in 1978 (9). This staging system has been modified, but can be broadly defined as suspected NEC (stage 1), definitive NEC (stage 2), severe NEC (stage 3). We have now modified the general classification of patients with NEC further based upon clinical presentation, as described below.

### Five clinical presentations of NEC in premature infants

**Figure 1.**
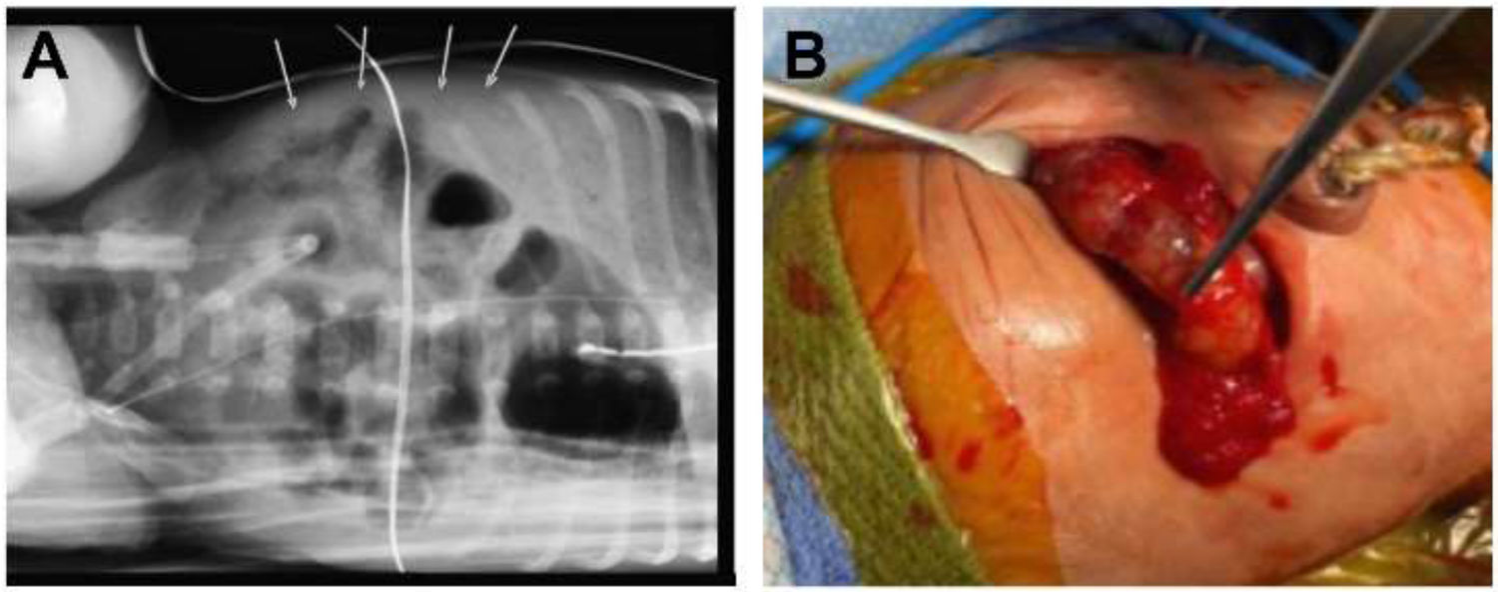
Radiologic and clinical findings in necrotizing enterocolitis. **A.** Abdominal x-ray revealing the presence of pneumatosis intestinal and intestinal dilation in a child with NEC; **B.** The intestinal findings in the patient in A, revealing the presence of intestinal ischemia and necrosis.

In view of the heterogeneity of clinical presentation of patients with NEC, we have recently described five different types of presentation, an approach which may allow for a greater understanding of the pathogenesis as well as determination of optimal treatment approaches. The first presentation refers to “*Textbook NEC” or “Classical NEC”* in which infants present initially with instability and abdominal tenderness, and then display radiographic changes of *pneumatosis intestinalis*, which is defined as air within the wall of the bowel (Figure 1A). After initial treatment, which typically includes the administration of broad-spectrum antibiotics and cessation of oral feeds, patients with *Textbook/Classical NEC* progress towards perforation, which reflects full thickness necrosis of the intestinal wall (Figure 1B). Infants with this presentation will require surgery, which typically involves resection of the necrotic intestine and creation of a stoma (10-12).

The second group of children present with “*medical NEC that fails to improve”* and reflects the group of children who have definitive NEC (*pneumatosis intestinalis*, abdominal tenderness) without the presence of pneumoperitoneum. These children benefit from surgery should they fail to improve within a defined period of time. Importantly, while the abdominal inflammation is ongoing, patients in this category are recognized to experience the effects of sustained inflammation on the developing brain (13), suggesting the benefit of a more aggressive surgical timeline and approach. The third group of patients refers to the group of infants who have “*abdominal tenderness and the presence of portal venous air”*, without the presence of pneumoperitoneum to signify that intestinal perforation has occurred. This group of patients is likely to have significant intestinal necrosis and a high anerobic bacterial load from which the portal venous air is derived, and for this reason, this group should be managed with exploratory laparotomy, similar to patients who have pneumoperitoneum. The fourth group of patients have “*staccato NEC”*, in which patients progress from mild signs of NEC to overwhelming sepsis with significant abdominal distention and bloody stools within a matter of hours. Expeditious laparotomy is indicated, occasionally allowing for the placement of an abdominal silo to allow for decompression of the abdominal contents and resection of clearly necrotic bowel. The fifth group of children exhibits the most severe form of NEC, namely “*NEC totalis”*, in which the vast majority of the small and large intestine is completely necrotic. In the past, infants with *NEC totalis* were left without further surgical treatment (14, 15) , yet, given the current positive results of intestinal rehabilitation programs and the success achievable with intestinal transplantation (16-18), an aggressive surgical approach may be warranted.

As can be seen, NEC is a heterogenous condition with at least five varying patterns of presentation. In the ensuing paragraphs, we present a unified approach to the pathogenesis of this devastating disorder, in order to identify novel therapeutic approaches for these children.

## The critical role of toll like receptor 4 (TLR4) in the Pathogenesis of NEC

To understand the pathogenesis of NEC, we developed clinically relevant animal models based upon a group of consistent clinical observations, which allowed us to generate a unifying hypothesis for this disease (19-23). As mentioned above, NEC develops most commonly in premature infants, after the administration of formula feeds, and generally after colonization of the gastrointestinal tract with enteric microbes (24). These observations led us to hypothesize that the interaction between feeds and intestinal microbes with an underdeveloped intestinal epithelium could play a major role in the pathogenesis of NEC.

**Figure 2.**
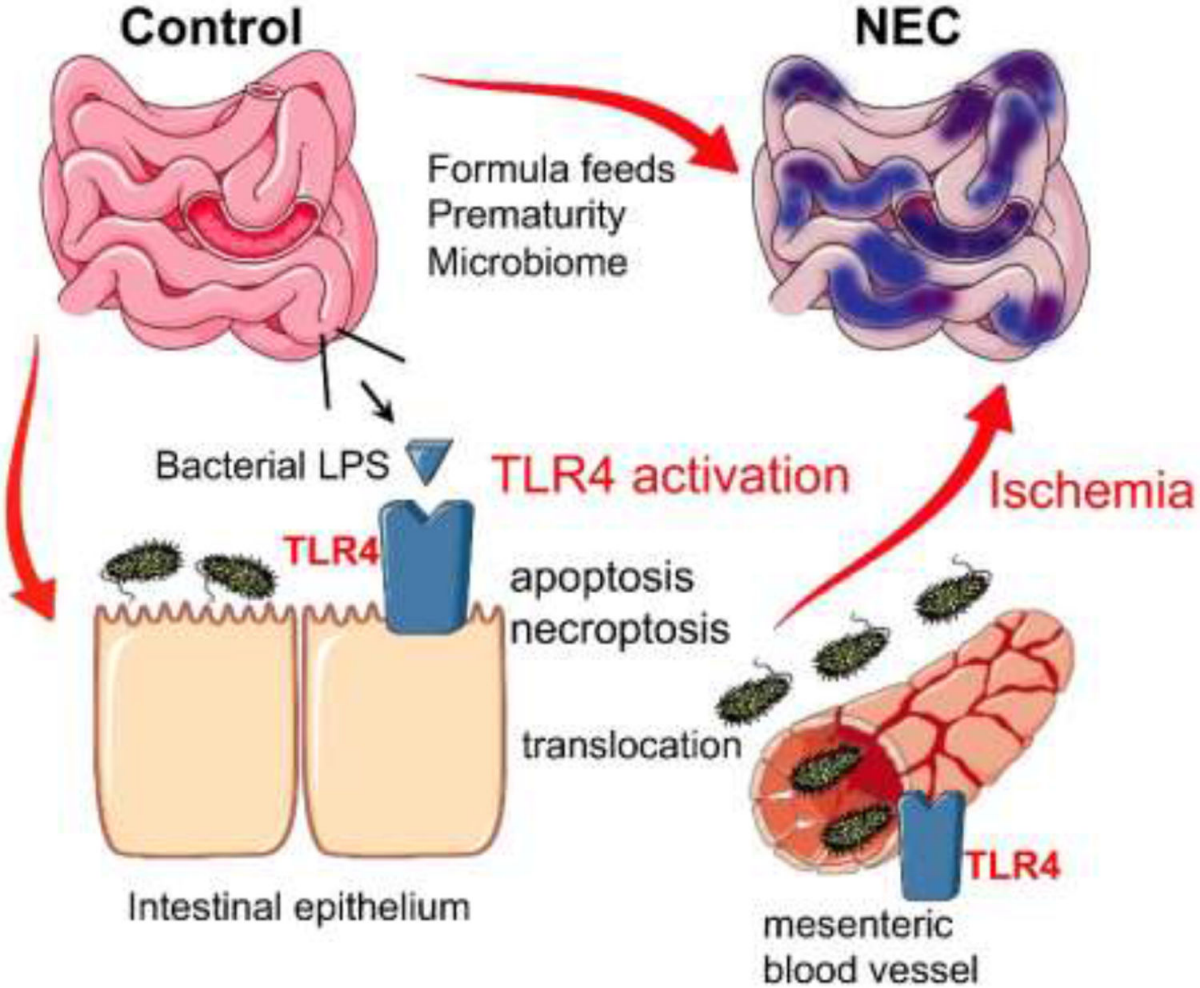
The role of TLR4 in the pathogenesis of NEC. Schematic depiction of the role of TLR4 signaling in NEC pathogenesis. As described in the text, in the setting of formula feeds, prematurity and a dysbiotic microbiome, intestinal TLR4 is activated by luminal bacteria. This activation results in apoptosis and necroptosis of the intestinal epithelium, resulting in bacterial translocation and TLR4 activation on the mesentery, leading to vasoconstriction, ischemia, and the development of NEC.

In order to test this hypothesis directly, we first explored the potential mechanisms by which intestinal bacteria could be recognized by the premature host, and whether such bacterial recognition pathways could be implicated in NEC development. These studies revealed the importance of toll like receptors (TLRs) on the intestinal epithelium in the pathogenesis of NEC (19, 21, 23) and Figure 2. TLRs are bacterial receptors that have major roles in the recognition and response by the host to invading organisms (25). The intestines of premature infants who develop NEC become colonized with TLR4-rich gram-negative bacteria, which suggested a role for the cognate gram-negative bacterial receptor, toll like receptor 4 (TLR4), in NEC pathogenesis (26-28). In support of this possibility, the expression of TLR4 was significantly elevated in the intestines of humans with NEC compared with infants without NEC (29, 30), raising the possibility that TLR4 activation on the intestinal epithelium could mediate NEC development (31-34). In order to test this possibility directly, we developed mice that lack TLR4 on the intestinal epithelium, which we then subjected to an experimental model of NEC that mimics the clinical conditions (19). Specifically, 1 week old mice (which are genetically and anatomically comparable with 28-week premature human infants (35) were administered “off-the-shelf” infant formula along with stool from an infant with severe NEC, five times per day, and were subjected to twice daily episodes of brief hypoxia, to mimic the apneic episodes seen in premature infants (36-39). After 4 days of treatment, mice that expressed TLR4 developed severe NEC, characterized by intestinal inflammation and necrosis (19, 40), while mice lacking TLR4 in the intestinal epithelium were significantly protected from NEC (19). These findings supported the initial hypothesis and revealed a critical role for intestinal TLR4 signaling in response to bacteria in the pathogenesis of NEC (41), and have been confirmed by laboratory groups around the world (22, 42-44).

Further studies have revealed the importance of TLR4 activation in causing NEC in humans. Specifically, Sampath *et al.* have shown that activating mutations in the TLR4 signaling pathway is linked to increased risk for NEC development in human infants (31, 45). Moreover, we and others have shown that the administration of breast milk feeds prevent the development of NEC in part through the inhibition of TLR4 signaling, which occurs through their component growth factors as well as through component oligosaccharides (46-48). Importantly, in seeking to identify novel treatments for NEC, we developed a family of small molecule inhibitors of TLR4, with an excellent safety profile (49). Our lead compound, termed C34, inhibited TLR4 and reduced inflammation in human tissue samples *ex vivo* that were resected from infants with surgical NEC. Taken together, these findings support a critical role for bacterial signaling through TLR4 in the pathogenesis of NEC in humans.

Having identified a critical role for TLR4 in the pathogenesis of NEC, we next sought to understand how TLR4 activation causes the intestinal injury and ischemia that are seen in NEC. In determining how TLR4 activation in the intestinal epithelium by luminal bacteria causes NEC, we and others have shown that TLR4 activation leads to a disruption in the normal balance between intestinal injury and repair (38, 50-52). Specifically, TLR activation on the intestinal epithelium leads to intestinal epithelial cell death through apoptosis (21, 50, 51) and necroptosis (38), while also inhibiting the mucosal repair mechanisms of intestinal epithelial migration and proliferation (29, 52). The subsequent injury to the intestinal mucosal barrier leads to bacterial translocation, after which bacteria can activate TLR4 on the lining of the mesenteric endothelium (41). Importantly, TLR4 signaling on the endothelium causes a dramatic and immediate vasoconstrictive response (53) due to reduced endothelial nitrate production (54). At the same time, TLR4 signaling in the newborn intestinal mucosa leads to the accumulation of proinflammatory Th17 lymphocytes at the expense of anti-inflammatory regulatory T (T_regs_) cells (55-57), which worsen the degree of inflammation and elicit the systemic septic response that is seen in patients with NEC (55, 58). Accordingly, strategies to reverse the effects of Th17 cells, or to block their influx into the intestinal mucosa, can reduce the degree of mucosal inflammation in NEC (55, 59). Along these lines, strategies to prevent the loss of endothelial nitric oxide synthase (eNOS) and to enhance vasodilation in the premature intestinal mesentery restores perfusion and limits intestinal ischemia in NEC (54). As of now, these strategies of interfering with these NEC inducing pathways have only been evaluated in animals (54, 55), but their early successes in preclinical studies suggest a potential therapeutic benefit for infants with NEC.

### An endogenous role for TLR4 in intestinal physiology

Having shown the importance of TLR4 signaling in the pathogenesis of NEC, it was also critical to understand whether there could be an endogenous role for TLR4 in normal intestinal physiology, which then could be rewired to lead to NEC. In support of this notion, we have shown that the expression of TLR4 in the intestinal mucosa rises during normal gut development in utero, and then falls at the time of full-term gestation to nearly undetectable levels (19, 21, 60). The reason for the elevated *in utero* expression of TLR4 reflects its non-immune role in the differentiation of intestinal stem cells and thus the regulation of normal intestinal development (20). The elevated expression of TLR4 in the premature gut explains in part the reasons for which premature infants are at higher risk for NEC development as compared with full term patients, in whom the levels of TLR4 have already decreased (25). Furthermore, prior studies have shown an inverse relationship between gestational age and the length of time it takes for NEC to develop i.e. that the more premature an infant is, the longer it takes for NEC to develop (61). This inverse relationship may be attributable in part to the fact that the expression of TLR4 in the developing intestine peaks at a fixed time point, and then falls at around the time of a full-term gestation (55, 60). Given that the fetus develops in a relatively sterile environment (62), the rising expression of TLR4 occurs without the induction of inflammation, whereas in the NICU, upon colonization of the host by enteric organisms, the developmental role for TLR4 switches to an inflammatory role, leading to the induction of NEC (25). It is also noteworthy that large clinical studies have shown that in patients who develop NEC, there is an accumulation of gram-negative bacteria that are richly endowed with TLR4 ligands (30, 63), providing further clinical support for the role of the bacterial receptor TLR4 in NEC pathogenesis in patients.

## Other contributing factors to the pathogenesis of NEC:

NEC development requires the activation of TLR4 in the intestinal epithelium, which leads to an imbalance between increased intestinal epithelial injury and reduced mucosal repair (29, 64). These research findings have occurred in parallel with additional studies from a variety of labs that have demonstrated the importance of a dysbiotic microbiome as well as an immature immune system in NEC pathogenesis, as detailed below:

### 1. Microbial dysbiosis in the pathogenesis of NEC.

A variety of studies have demonstrated a difference and lack of diversity in the microbiota of patients who develop NEC as compared with patients who do not. These differences include an increase in Proteobacteria (particularly Enterobacteriaceae) and a decrease in Firmicutes and Bacteroidetes relative to healthy infants (26-28), which are rich in TLR4 ligands, and thus capable of inducing NEC (27). Several factors can contribute to the changes in the intestinal microbiome that is acquired in the newborn intestine, including mode of delivery, type of feeding, and antibiotic exposure (65, 66). For instance, vertical transmission of bacterial colonies was found to be related to the mode of delivery as newborns who were delivered vaginally harbored gut bacteria comparable to the mother’s vaginal microbiome, whereas newborns who were delivered via Cesarean section harbored bacteria similar to the mother’s skin microbiota (66). After the initial exposure to mother’s bacterial colonies, the ongoing colonization of the newborn intestine can be further influenced by feeding type. Breast fed infants are found to have intestinal colonies dominated by Bifidobacteria and Bacteroides which may have physiologically relevant benefits, such as improved modulation of the immune system, vitamin production, and decreases the risk of allergic disease (67, 68). This finding may explain why the routine use of perinatal antibiotics in preterm infants can increase the risk for NEC development (69-71).

Given the potential importance of the intestinal microbiome in the pathogenesis of NEC, several groups have sought to manipulate the microbiome through the use of probiotics in order to prevent NEC (72-74). Probiotics are defined as living organisms that provide the host with health benefits when ingested (75). Investigations into the protective mechanism of probiotics on the intestine have demonstrated a downregulation of pro-inflammatory genes (76, 77), competition with pathogenic microbes (78), and production of butyrate which serves as a fuel to the intestinal epithelium (79, 80). The evidence in support of a role for probiotics in the prevention of NEC includes a large meta-analysis of over 10,000 infants, which identified a reduction in the incidence of severe NEC with the use of probiotics (Probiotics 170 cases vs Control 311 cases, RR 0.53 [0.42-0.66]) (81). However, not all studies have reached the same conclusion. For instance, the Probiotics in Very Preterm Infants (PiPS) trial did not find any difference in Bell Stage ≥ 2 NEC risk after treating 1,315 infants with probiotics or placebo (Probiotics 9.4% vs Placebo 10.0%, RR 0.93 [0.68-1.27]) (82). In summary, while probiotics are likely to have a beneficial role in certain infants who are at risk for NEC development, lingering questions remain regarding the safety and efficacy of probiotic preparations, which together have limited the overall acceptance of these reagents in babies at risk for NEC.

### 2. The role of the maternal diet in NEC development: the aryl hydrocarbon receptor pathway

Given the importance of diet on the pathogenesis of NEC, we and others have focused on potential dietary receptors that could mediate disease development. In this regard, we have shown a therapeutic potential for the aryl hydrocarbon receptor (AHR), and its dietary ligand indole-3-carbinol (I3C) (83). I3C is found in green leafy vegetables and human breast milk and was shown to protect the intestinal epithelium of mice and humans through a reduction in TLR4 expression and signaling (83). Interestingly, feeding mice with an AHR ligand during pregnancy significantly downregulated TLR4 signaling and reduced NEC in the offspring, indicating that maternal dietary intervention could reduce NEC severity (83). Moreover, these studies also led to the identification of a novel AHR ligand that could reduce NEC, while also explaining how a maternal diet rich in endogenous AHR ligands, such as the Mediterranean diet, can reduce the incidence of NEC (84).

### 3. Impaired intestinal motility in the pathogenesis of NEC

One of the earliest findings seen in patients with NEC is the development of abdominal distention and vomiting, which together reflect impaired intestinal motility and the presence of ileus (85, 86). The presence of ileus in patients with NEC has long been considered a *consequence* of the diagnosis, which is thought to reflect injury to the enteric nervous system after bowel ischemia has occurred (87). Recently we challenged this dogma and showed instead that the development of intestinal dysmotility reflects an upstream *cause* rather than a *consequence* of NEC (88). Specifically, in mice and humans, NEC was associated with a loss of intestinal motility that was mediated by a reduction in the enteric glia (88). In addition to providing support for the neurons within the intestine, the enteric glia also were found to serve an anti-inflammatory role, through the release of the molecule brain-derived neurotrophic factor (BDNF), which normally acts to restrain TLR4 signaling on the overlying intestinal epithelium (88). These findings led to our novel pre-clinical strategy of attempting to restore enteric glial activity – using a newly discovered small molecule glial activator, called J11, to reduce NEC severity (88). Moreover, the administration of prokinetic agents Cisapride and Metoclopramide increased motility and reduced NEC severity in mice (88), adding additional proof-of-concept to this strategy. Taken together, these findings suggest that novel therapeutic approaches based upon improving intestinal motility could prevent the progression of NEC and improve outcomes.

## Consequences of NEC on the developing brain

The above studies reveal the important pathways by which bacterial signaling in the intestinal epithelium leads to the development of NEC through TLR4 activation, and they have shown that interference with these signaling pathways can reduce NEC severity in preclinical models. Recently, there has been significant interest in seeking to optimize the long-term health of patients who survive NEC in infancy (89, 90). To this end, it has also become increasingly recognized that NEC survivors suffer severe neurocognitive impairment as they get older (91-93), and that they display delayed learning and impaired social interactions as compared with their gestational age-matched peers who did not develop NEC (92). At the structural level, the brain MRIs of children who recover from NEC reveal persistent white matter injury, particularly in the periventricular and prefrontal regions, areas that are associated with learning and memory (94). In seeking to understand the mechanisms that may explain the pathophysiology of NEC-induced brain injury, we have recently identified a critical role for the immune cells of the brain, also called the microglia, whose activation is critical in causing the white matter and neurocognitive impairment (95). In terms of the mechanisms involved, TLR4 activation on the microglia occurred in response to the release of the pro-inflammatory microglial ligand, high mobility group box 1, (HMGB1) from the inflamed intestinal mucosa (95). Strategies to prevent the release of reactive oxygen species from the activated microglia resulted in a reduction of white matter injury and a reversal of neurocognitive impairment in mice (95). In follow on studies, we also showed that IFNγ-releasing lymphocytes exit the intestine and accumulate in the inflamed neonatal brain in the setting of NEC, compounding the white matter injury (13). Remarkably, the inhibition of these intestinal-derived lymphocytes prevented the white matter injury in mice (13). These findings may have significant implications for how we manage infants with NEC. In other words, the finding that pro-inflammatory cells and molecules that exit the inflamed intestine in NEC to cause white matter injury suggests that the longer the intestinal inflammation occurs, the greater the brain injury that accompanies it. In view of these findings, we recommend that the impact of inflammation on the developing neonatal brain be considered in the surgical decision making of patients with severe NEC needing surgery.

## Conclusions

NEC remains the leading cause of mortality from gastrointestinal disease in premature infants. In providing a unifying model to explain the pathogenesis of NEC, we have shown that TLR4 activation in response to intestinal microbial dysbiosis leads to mucosal injury leading to microvasculature vasoconstriction, which in association with a loss of enteric glia, results in the devastating intestinal damage seen in NEC. Accordingly, the inhibition of TLR4 and its downstream signaling molecules has been shown to prevent or to treat NEC in preclinical studies, while strategies to enhance enteric nervous system activity can attenuate NEC in preclinical models. More recently, the administration of a maternal diet that is rich in AHR ligands can reduce the exaggerated TLR4 signaling in the offspring and reduce NEC. Finally, strategies to limit intestinal-derived pro-inflammatory molecules and cells can reverse the devastating neurocognitive impairment and structural damage seen in the setting of NEC in preclinical models and suggest that earlier surgical resection of necrotic intestine may reverse NEC-induced brain injury. Ongoing studies in the field will seek to further expand our knowledge and understanding of this disease and improve the care for patients diagnosed with NEC.
